# Coracoid fractures: long-term results and modification of the classification

**DOI:** 10.1007/s00590-025-04493-0

**Published:** 2025-09-04

**Authors:** Johanna Habarta, Christian Färber, Martin Jordan, Fabian Gilbert, Rainer Meffert, Jonas Schmalzl

**Affiliations:** 1 Department of Trauma, Hand, Plastic and Reconstructive Surgery, University Hospital Julius, Maximilians-University, Oberduerrbacher Straße 6, 97080 Würzburg, Germany; 2 Trauma, Reconstructive Surgery, and Rehabilitation Medicine, University Hospital University Greifswald, Fleischmannstraße 8, 17475 Greifswald, Germany; 3 Department of traumatic surgery and orthopedics, reconstructive surgery, sports surgery, Kreisklinik Ebersberg, Pfarrer-Guggetzer-Str., 85560 Ebersberg, Germany

**Keywords:** Coracoid fracture, Acromioclavicular joint dislocation, Glenoid fracture, Classification

## Introduction

Coracoid fractures are rare injuries of the upper extremity [1–10] with a reported occurrence of 3–13% of all scapular fractures [1, 3–6, 8–11]. The most common injury mechanisms are traffic accidents, falls from height and sports injuries [1, 2, 5, 7, 9–18]. The coracoid process is a bony process [19] of the scapula and is part of the superior shoulder suspensory complex (SSSC), an union of bone and soft tissue structures which provide the stability of the shoulder girdle. The SSSC consists of the coracoid process, the acromion, the acromioclavicular joint (ACJ), the coracoclavicular (CC) ligaments, the glenoid fossa and the clavicle. It is well known that single injuries of the SSSC do not seriously impact stability of the shoulder girdle, whereas multiple disruptions can lead to unstable injuries and need particular attention in treatment [1, 2, 4, 7–14, 16, 19, 19, 20]. Coracoid fractures frequently come along with concomitant injuries [1, 2, 4, 5, 7, 9, 10, 12, 13, 15, 19, 21], such as ACJ dislocations [1, 2, 6–8, 10, 12–14, 16–18, 20–23], glenohumeral dislocations [1, 2, 4, 10] and accompanying fractures of the scapula and the clavicle [1, 2, 4, 10, 12, 15, 21]. Due to its anatomical position, on radiographs the coracoid process presents as an overlay structure to the humeral head and the glenoid rim. That is why diagnosis of coracoid fractures is crucial and fractures may often be overlooked, in particular if the fracture fragment is not or only slightly displaced [1–6, 10, 13–17, 19]. Treatment of coracoid fractures is controversial and has to be defined individually. Both, conservative and surgical procedures are possible options [1–4, 6–17, 19, 22, 23]. Slightly displaced and non-displaced fractures may successfully be treated conservatively and rarely require surgical treatment. Surgical treatment may be chosen in case of double or multiple disruptions of the SSSC [1, 2, 4, 7, 8, 10–14, 16, 19, 19, 20], fragment displacement of more than 1 cm (cm) or patients with high physical strain on the upper extremities or overhead and throwing athletes [10, 12, 15, 20, 22]. Coracoid fractures often show very good clinical outcomes [1, 4, 6–8, 10, 12–14, 16, 18–20, 22, 23] and surgical and non-surgical treatment seems to be comparable in many cases [1, 3–5, 10, 12, 17]. Overall, complications are rather low. Relatively common complications are coracoid nonunion [1, 4, 7, 11, 12, 15], which mostly go along with conservative treatment [2, 4]. Nonetheless, nonunion is not always symptomatic [4, 16] and in those cases, further treatment is not necessary [2, 16]. Currently, there are two commonly used fracture classification systems for coracoid fractures, the Ogawa and the Eyres classification. The Ogawa system differs between two types: Type I is a proximal fracture near to the coracoid base and Type II is a distal fracture of the coracoid apex without involvement of the CC ligaments [4–6, 10, 11, 13–16, 18, 19, 21]. The Eyres system differentiates 5 types, depending on the localisation at the coracoid [4, 5, 7, 10, 11, 13, 15, 16, 21]. However, neither of them include concomitant injuries. The aim of this study was to review all cases of the last 12 years in a level 1 trauma centre and to present an expanded CT-based fracture classification system based on biomechanical considerations taking into account secondary injuries.

## Materials and methods

This clinical trial is a retrospective case series of coracoid fractures in a level 1 trauma centre over a period of 12 years. Patient records, radiographs, computed tomography (CT) images and the clinical outcome were evaluated. All patients who received a treatment in our hospital with a coracoid fracture in this period were contacted to participate in our study. Exclusion criteria were pathological fracture. Fracture classification was performed according to the Ogawa and Eyres classification systems. The data were collected after a signed declaration of consent. A positive vote from the academic ethics committee is available. The follow-up examination respect the ethical standards in the Helsinki Declaration of 1975, as revised in 2000, as well as the national law. At the follow-up examination, the range of motion was measured with a goniometer and muscle strength with a dynamometer. To objectify everyday abilities and life quality, the following scores were assessed: American Shoulder and Elbow Surgeons Score (ASES), the Constant-Murley Score (CMS), the Subjective Shoulder Value (SSV) and the quick Disabilities of the Arm, Shoulder and Hand Score (qDASH). In addition, complication and revision rate, and in case of surgical treatment the surgical technique and approach were recorded. Surgical techniques that were used were screw osteosynthesis, K-wire transfixation and lateral hook plate. Fracture consolidation was evaluated on scheduled follow-up radiographs or CT images. All data were collected and analysed with Microsoft Excel Version 2016.

## Results

Out of 51 cases, 15 patients with a coracoid fracture were available for follow-up and could be included in the study. Four underwent surgical treatment and 11 were treated conservatively. Among the surgically treated patients, all of the patients had fractures proximal to the CC ligaments. Two fractures were treated with screw osteosynthesis and 1 patient with an accompanying ACJ dislocation received a hook plate. Therefore, in 2 cases open surgery was performed, 1 case was treated arthroscopically assisted. One patient underwent secondary surgery due to a symptomatic pseudarthrosis of the coracoid tip. The study cohort consisted of 5 women and 10 men and the mean age was 46 years (SD 16). The mean follow-up period amounted to 65 (SD 41) months (range 6–150 months). 8 injuries (53 per cent) affected the dominant arm. Fracture classification was performed according to the common Ogawa and Eyres classification systems. In total there were 2 Eyres type I, 4 type III, 5 type IV and 4 type V fractures. Applying the Ogawa classification there were 13 type I and 2 type II fractures. All data are shown in Table 1.

**Table 1:** Patients demographics

Variable	N [%]
*Treatment*	
Conservative Operative	11 [73] 4 [27]
*Gender*	
M F	10 [67] 5 [33]
*Age in years [SD]*	
Mean Mean conservative Mean operative	46 [± 16] 51 [± 13] 32 [± 15]
*Fracture classification*	
Ogawa type 1 Ogawa type 2 Eyres type 1 Eyres type 2 Eyres type 3 Eyres type 4 Eyres type 5	13 [87] 2 [13] 2 [13] 0 [0] 4 [27] 5 [33] 4 [27]
*Time to follow-up in months [SD]*	
Total Conservative Operative	65 [± 41] 70 [± 45] 53 [± 31]
*Injury side*	
Non-dominant limb Dominant limb Thereof operative treatment	7 [47] 8 [53] 2 [13]
*Requirement of pain medication*	
Conservative Operative	3 [20] 0 [0]

### Follow-up results

The following results were recorded during the follow-up examination and are presented in Table 2. Rest pain was determined based on the VAS and was 0.8 (SD 1.0) in the surgical group and 0.7 (SD 1.3) in the conservative group. Three patients of the conservative group still required pain medication, but none of the operative group. The SSV was 74 (SD 29) in the conservative and 74 (18) in the operative group. The ASES valued 80 (SD 22) in the conservative versus 81 (SD 13) in the operative group. The CMS and the qDASH showed slightly better results among the operative patients. The CMS was 75 (SD 21) in the conservative group versus 79 (SD 16) in the operative group. The qDASH amounted for 25 (SD 28) in the conservative versus 17 (SD 14) in the operative group. The measured ranges of motion are shown in Table 3.

**Table 2:** Outcome values

Variable	Total	Conservative	Operative
*VAS [SD]*			
Mean	0.8 [1]	0.7 [1]	0.7 [1]
*ASES [SD]*			
Mean Mean non-injured limb	80 [22] 99 [3]	80 [22] 99 [3]	81 [13] 98 [4]
*SSV [SD]*			
Mean	70 [26]	74 [29]	74 [18]
*Constant score [SD]*			
Mean	76 [20]	75 [21]	79 [16]
*Quick DASH score [SD]* Mean	23 [25]	25 [28]	17 [14]
*Weight in N [SD]*			
Mean Mean non-injured limb	75 [50] 100 [50]	74 [54] 99 [41]	79 [43] 103 [78]

**Table 3:** Range of motion

Mean range of motion (°) [SD]	Total	Total non-injured	Conservative	Conservative non-injured	Operative	Operative non-injured
Abduction	137 [37]	155 [34]	135 [35]	157 [27]	143 [48]	150 [54]
Adduction	25 [11]	29 [11]	26 [10]	31 [9]	23 [15]	23 [15]
Flexion	139 [47]	166 [16]	131 [4: 9]	164 [16]	160 [40]	170 [20]
External rotation	50 [23]	54 [24]	48 [23]	53 [18]	54 [26]	58 [40]
Internal rotation	Th12	Th11	Th12	Th11	LWK2	Th11
Retroversion	42 [18]	55 [12]	42 [17]	53 [14]	43 [22]	61 [3]
External rotation in 90°	67 [27]	77 [25]	62 [28]	81 [16]	80 [20]	68 [45]
Internal rotation in 90°	48 [21]	56 [16]	49 [18]	55 [16]	46 [29]	56 [19]

### Complications

Four complications occurred among 3 patients who were treated non-surgically. One patient developed a symptomatic nonunion and suffered from chronic pain and limited range of motion. One patient with an accompanying acromion and lateral clavicle fracture (i.e. a triple disruption of the SSSC) could not undergo operative treatment due to severe previous illnesses and developed a nonunion of the coracoid, the acromion and the clavicle. The third suffered from chronic severely limited range of motion with an abduction of 120°. In one patient a displaced coracoid tip fracture after a shoulder dislocation was overseen and this patient developed a nonunion with painful limited range of motion. Consequently, she was treated surgically with arthroscopic labral repair and pseudarthrosis resection and refixation of the coracoid tip with suture anchors. Thus, this patient was included in the operative group. In the operative group the patient who was treated with a hook plate underwent elective implant removal, following our standard protocol to prevent secondary damage of the acromion.

### Concomitant injuries

All patients had concomitant injuries. These included 6 glenoid fractures, 3 acromion fractures and 4 ACJ dislocations. All concomitant injuries are presented in Table 4.

**Table 4:** Concomitant injuries

Injury	N
Glenoid fracture	6
Fracture of other part of the scapule	6
AC joint dislocation	4
Rip fracture	4
Clavicle fracture	3
Acromion fracture	3
Shoulder dislocation	4
Nerve damage	2
Spine/extremity fracture	2
Head or facial trauma	2
Humerus fracture	1
Total	37

## Discussion

Aim of this study was to analyse coracoid fracture patterns, their complications and concomitant injuries and to discuss treatment options in context of the collected data and the available literature. It should be noted that, due to the rarity of the fracture, the available literature often has low levels of evidence [10]. Among the patients, most had Ogawa type I fractures (87%) at the coracoid base and according to the Eyres classification, most cases were type III-V (87%). Thereby, the results of the study presented correspond with those of previous studies, which report of mainly base-near fractures. In young patients and children, it must be kept in mind that fusion of the coracoid bone is often completed very late, and an open growth plate can be misinterpreted as a fracture [10]. Ogawa type I fractures are reported from 77 to 100% [1, 4, 7, 11, 13, 18]. Most patients (67%) were men and the mean age was 46 (SD 16) years. In prior studies patients also predominantly were male (78–100%) [1, 2, 5, 6, 8, 11, 13, 16, 17, 21–24] and the mean age was reported from 20 to 55 years [2, 4, 6–8, 11, 13, 14, 16–18, 21–24]. This might be due to the fact that frequent injury causes are high energy trauma and sports injuries [1, 2, 5, 7, 9–17], which often affect younger and middle aged men. Knapik et al. report of a mean age of 19 years in a cohort of sports injuries [5].

All of our cases had concomitant injuries. The most common were glenoid fractures (40%), ACJ dislocations (27%) and other fractures of the scapula (60%). Ogawa et al. reported of concomitant ACJ dislocations in 33% and other scapula fractures in 15% [1]. Van Doesburg et al. found 216 concomitant injuries in 110 patients. ACJ dislocations accounted for 18%, glenoid fractures for 11% and other scapula fractures for 15% of the cases [4]. In patients with coracoid nonunion, Ogawa et al. reported of concomitant ACJ dislocations in 20% and anterior shoulder dislocation in 50% of the patients [2]. Hill et al. found 4 associated ACJ dislocations and 16 intraarticular glenoid fractures in their cohort of 22 patients [11]. In a cohort of patients with sports injuries, Knapik et al. found combinded ACJ dislocations in 9 of 21 patients and anterior glenoid fracture in 2 cases [5].

Treatment of coracoid fractures depends on certain conditions and both conservative and operative procedures should be considered [1–4, 7–17, 19]. In general after review of the literature operative treatment is recommended in case of specific conditions, such as fracture dislocation of the coracoid tip of more than 1 cm [10, 12, 15], a double disruption of the SSSC [1, 2, 4, 7, 8, 10–14, 16–19, 19, 20], patients with high functional demands or patients with concomitant glenoid fracture with an intrarticular displacement of more than 2 mm [10, 12]. In the examined cohort, 10 patients had double or multiple disruption of the SSSC, but due to the fact that most patients suffered a severe polytrauma, in these cases the coracoid fractures were considered of secondary importance and only 3 patients (25%) underwent surgical treatment. All of them three were fractures of the base of the coracoid. One had an accompanying ACJ dislocation, 1 had an ACJ dislocation and a glenoid fracture and the third had an accompanying displaced glenoid fracture. The patient who received secondarily operative treatment was a displaced coracoid tip fracture and had an accompanying shoulder dislocation. Regarding the clinical outcome, operatively treated patients showed slightly better results compared to the conservative group in our study cohort. This might also be due to the difference of mean age, which was 32 (SD 15) in the operative cases and 51 (SD 13) in the conservative group. Overall outcome showed satisfactory results for both groups. Previous studies reported of good to excellent results in the follow-up of isolated and non-isolated coracoid fractures [1, 4, 6–8, 10, 12–14, 16–19, 22–24] and results were comparable between conservative and operative treatment [1, 3–5, 10, 12, 17]. However, coracoid fractures are rare injuries and the available data are scarce and of limited quality as all articles are case series with low case numbers. Hill et al. reported of a mean DASH Score of 12 after a follow-up of 22 months in a cohort of 22 patients with only surgically treated patients [11]. In a review of a total of 197 cases by Ogawa et al., unsatisfactory results were reported in conservatively treated patients with multiple disruptions of the SSSC [1]. However, van Doesburg et al. recommended to consider conservative procedures even in some cases for double disruptions of the SSSC as a conclusion of their review including 110 cases [4]. Knapik et al. compared the return to sports rate in 21 cases with only sports injuries and showed no significant differences between surgically and conservatively treated patients [5].

As shown in our study and as reported in the literature [1, 2, 4, 5, 7, 9, 10, 12, 13, 15, 17–19, 21] nearly all patients present secondary injuries of the shoulder girdle, however neither the Eyres nor the Ogawa classification include associated injuries. In most patients who were treated operatively, surgery was performed to address multiple disruptions of the SSSC. To our knowledge, there is no clear consensus about the choice of treatment depending on the fracture pattern. In a synopsis of the results of our study, the available literature and biomechanical considerations, we suggest an upgraded fracture classification system for coracoid fractures in the following.

**Fig. 1:**
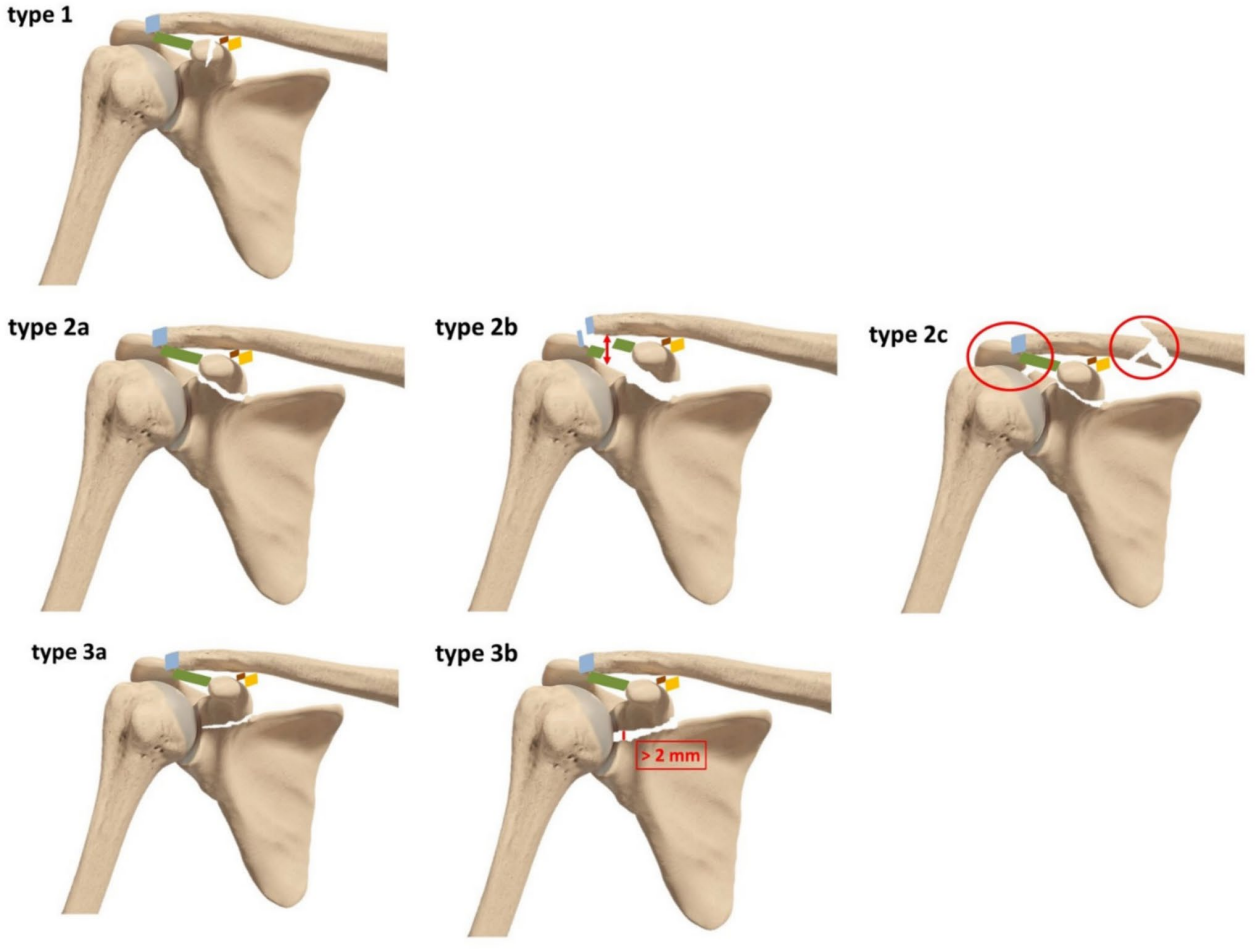
Fracture classification: Type 1: isolated, distal coracoid fracture; type 2a: isolated proximal coracoid fracture; type 2b: proximal coracoid fracture with combined AC joint dislocation; 2c: proximal coracoid fracture with concomitant clavicle and acromion fracture 3a: proximal coracoid fracture with non-displaced glenoid involvement; 3b: proximal coracoid fracture with an intraarticular fracture fap of more than 2 mm

First, a categorisation of the fractures according to their localisation in relation to the CC ligaments should be considered. A fracture proximal to the CC ligaments can cause far more instability than a distal fracture. For exact fracture classification, we highly recommend a CT scan with 3D reconstruction. Fractures distal to the CC ligaments were classified as type 1. An isolated fracture proximal to the CC ligaments without concomitant ACJ dislocation was classified as type 2a. In all patients with a fracture proximal to the CC ligaments additional bilateral Zanca stress radiographs should be performed to rule out an ACJ injury. A Zanca view is a modified anteroposterior (AP) radiograph of the shoulder that focuses on the AC joint. In a bilateral Zanca stress radiograph, the view is taken while the patient holds weights (typically 5–10 pounds) in each hand. This creates downward traction on the shoulders and exaggerates any instability or separation at the AC joint. Cases with concomitant ACJ dislocation with an increased AC distance should be graded as type 2b injuries. Cases with concomitant acromion and clavicle fractures should be graded as type 2c lesions. Coracoid fractures proximal to the CC ligaments affecting the glenoid fossa should be classified as type 3 lesions. A distinction should be made between type 3a fractures with no or minimal joint displacement of less than 2 mm and type 3b fractures with a gap of the joint line of more than 2 mm. The suggested classification system is illustrated in Fig. 1.

Type 1 fractures can usually be treated conservatively. In case of severe dislocation of more than 1 cm or high demand patients a screw osteosynthesis can be performed. We recommend to realise conservative treatment with sling immobilization for 2 weeks and early functional mobilization. However, flexion and supination against resistance should be avoided for the first 6 weeks after trauma. Radiographs are only recommended in cases of ongoing pain. In other studies, sling immobilization is maintained for 6 weeks, and mobilization begins only after this period has ended [10].

As mentioned above for type 2 lesions additional bilateral Zanca stress radiographs should be performed [9] in order to be able to differentiate between type 2a and 2b lesions. If the AC distance is normal, the fracture is classified as type 2a and conservative treatment is recommended with the same treatment protocol as for type 1 lesions.

For type 2b lesions operative treatment should be recommended as it is known, that double disruptions of the SSSC result in further instability with an increased risk of fracture nonunion [1, 2, 4, 7–9, 11–14, 16, 18, 19, 19, 20]. In the study presented, two cases that were treated conservatively due to incompliance and severe polytrauma showed undesirable results with limited range of motion and chronic pain. Combalia et al. reported in a case series of coracoid fractures combined with ACJ dislocations of similar results in conservative and operative treatment and recommend an individual evaluation of the case [17].

**Fig. 2:**
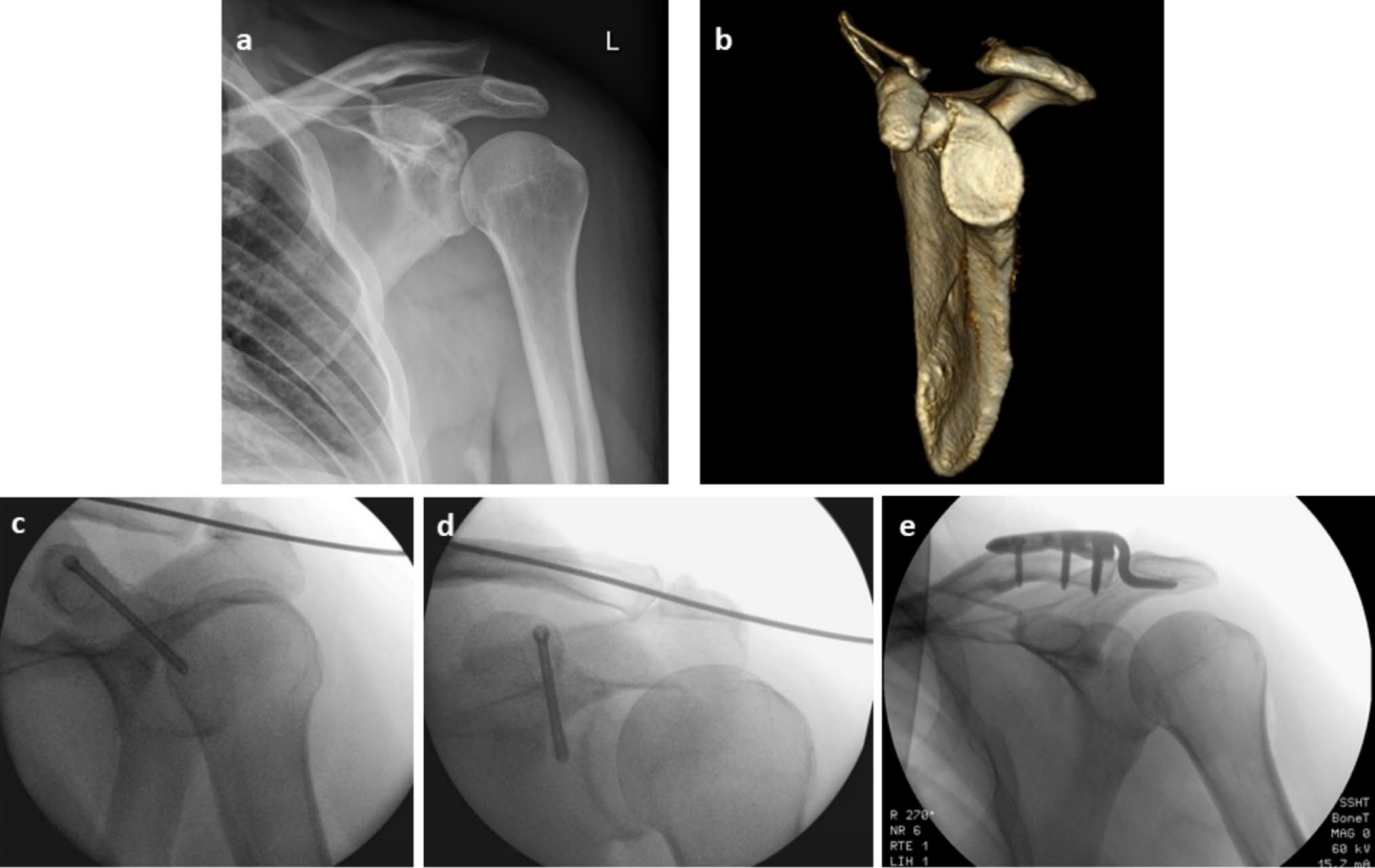
Coracoid base fracture with concomitant AC-joint dislocation (type 2b) **a**, **b**, **c** and **d** type 2b coracoid fracture treated with screw osteosynthesis and K-wire reduction in the AC joint; **e** type 2b coracoid fracture after hook plate osteosynthesis

There are 2 options for surgical care of type 2b lesions. Open reduction in the ACJ with a hook plate resulting in an indirect reposition of the coracoid fracture or K-wire transfixation of the ACJ in combination with an open or arthroscopically assisted screw osteosynthesis of the coracoid fracture. In this case series there was one 2b lesion with surgical treatment (hook plate). At follow-up the CMS was 84. Figure 2 shows pre- and postoperative radiological examinations for the 2 different surgical techniques. There are few reports of cases with a combined ACJ dislocation and coracoid fracture treated with a hook plate [6, 7, 13, 14, 18]. All patients showed very good clinical outcome parameters [6, 7, 14, 18], with full range of motion [6, 7, 14] and CMS of 92–99 [7, 14, 18]. Elshahhat et al. performed open reduction in coracoid fractures with associated ACJ dislocations using a hook plate in combination with a screw osteosynthesis for patients with high clinical demands. They perform reduction under intraoperative fluoroscopic control and report of satisfactory clinical results [18]. Performing an open reduction, an intraoperative fluoroscopic control can be helpful and should be considered in case of combined coracoid fracture and ACJ dislocation. Depending on the surgical method, one option is to first reduce the AC joint dislocation intraoperatively and then check the position of the coracoid. If the coracoid is well-positioned, surgical stabilization of the coracoid may be omitted [10]. Wilson et al. reported of a case with K-wire transfixation and screw osteosynthesis with regain of full range of motion and fracture consolidation at final follow-up [23]. Both procedures are to be regarded as equivalent and can be chosen according to individual preferences. A disadvantage of the hook plate is the need for implant removal under anaesthesia [6, 13, 14, 18], while K-wire removal can take place in local anaesthesia.

Type 2c lesions should be treated operatively as they represent a triple disruption of the SSSC, and therefore, conservative treatment cannot lead to sufficient stability. In our group there was one patient with this combined injury who was treated conservatively due to his previous illnesses. He developed a symptomatic nonunion.

**Fig. 3:**
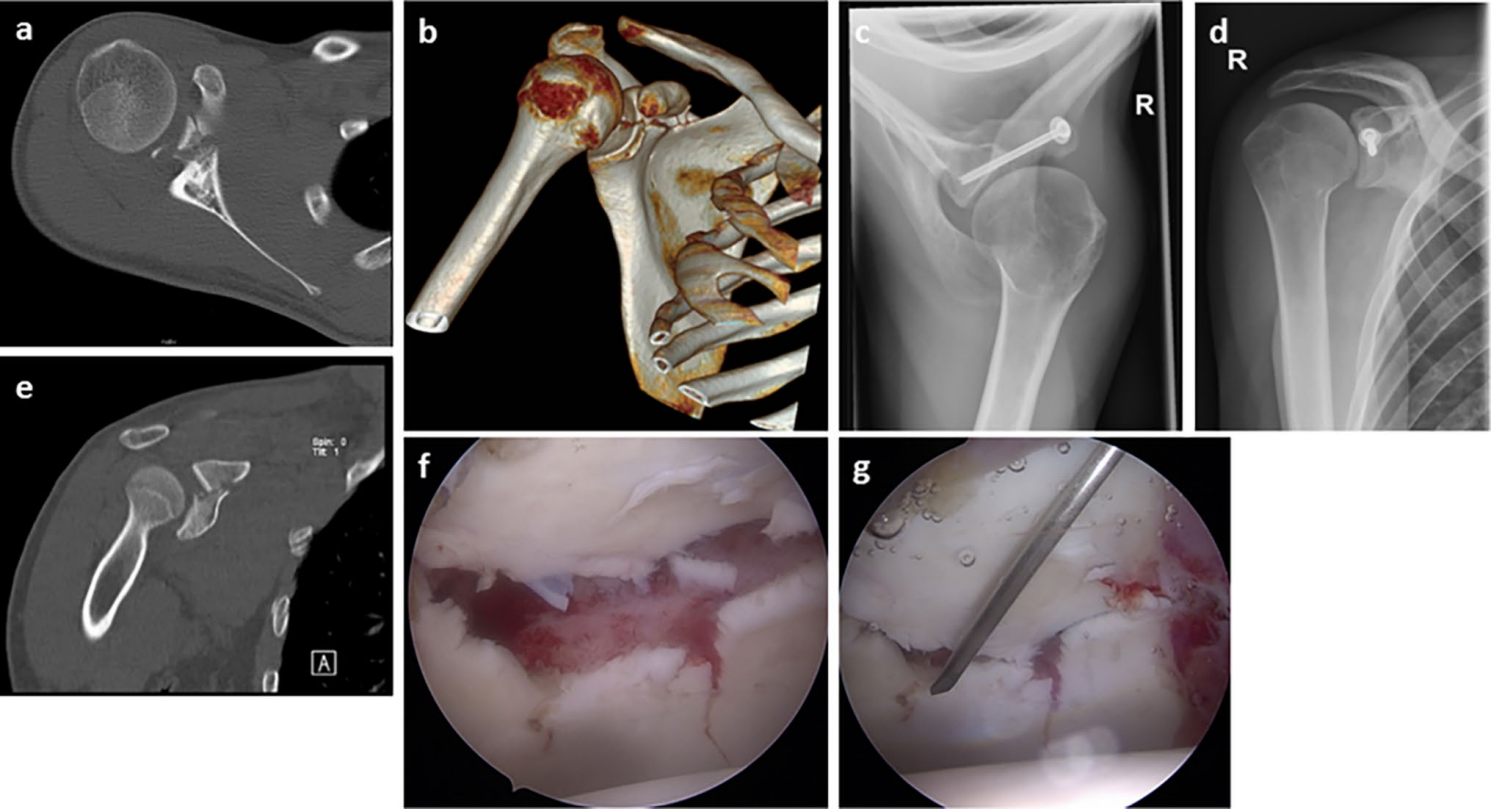
**a** and **e** CT images of a type 3b coracoid fracture; **b** 3D CT image of a type 3b coracoid fracture; **c** and **d** type 3b coracoid fracture with screw osteosynthesis; **f** and **g** intraoperative arthroscopic view on displaced glenoid fracture

Regarding type 3 injuries an exact analysis of the CT images should be performed. In cases of an intraarticular gap of more than 2 mm, we recommend an arthroscopically assisted osteosynthesis with cannulated screws as injuries of the joint surface should be reconstructed as good as possible [9]. The screw insertion can be performed either through the Neviaser portal or through an additional direct portal anterior to the coracoid process. The Neviaser portal is an additional portal medial to the acromion, typically through the supraspinatus fossa, i.e. the soft spot bordered by the acromion, clavicle, and scapular spine. Open reduction and screw osteosynthesis is also possible. Figure 3 shows pre- and postoperative images of a type 3b lesion. Goss et al. highly recommend surgical treatment of severely displaced glenoid fractures and suggest the use of plate osteosynthesis, interfragmentary compression screw osteosynthesis or cannulated screw osteosynthesis [9]. Wafasaide et al. reported of a case of a type 3b fracture that was treated with arthroscopically assisted osteosynthesis with a cannulated screw using an anterior portal [25].

**Fig. 4:**
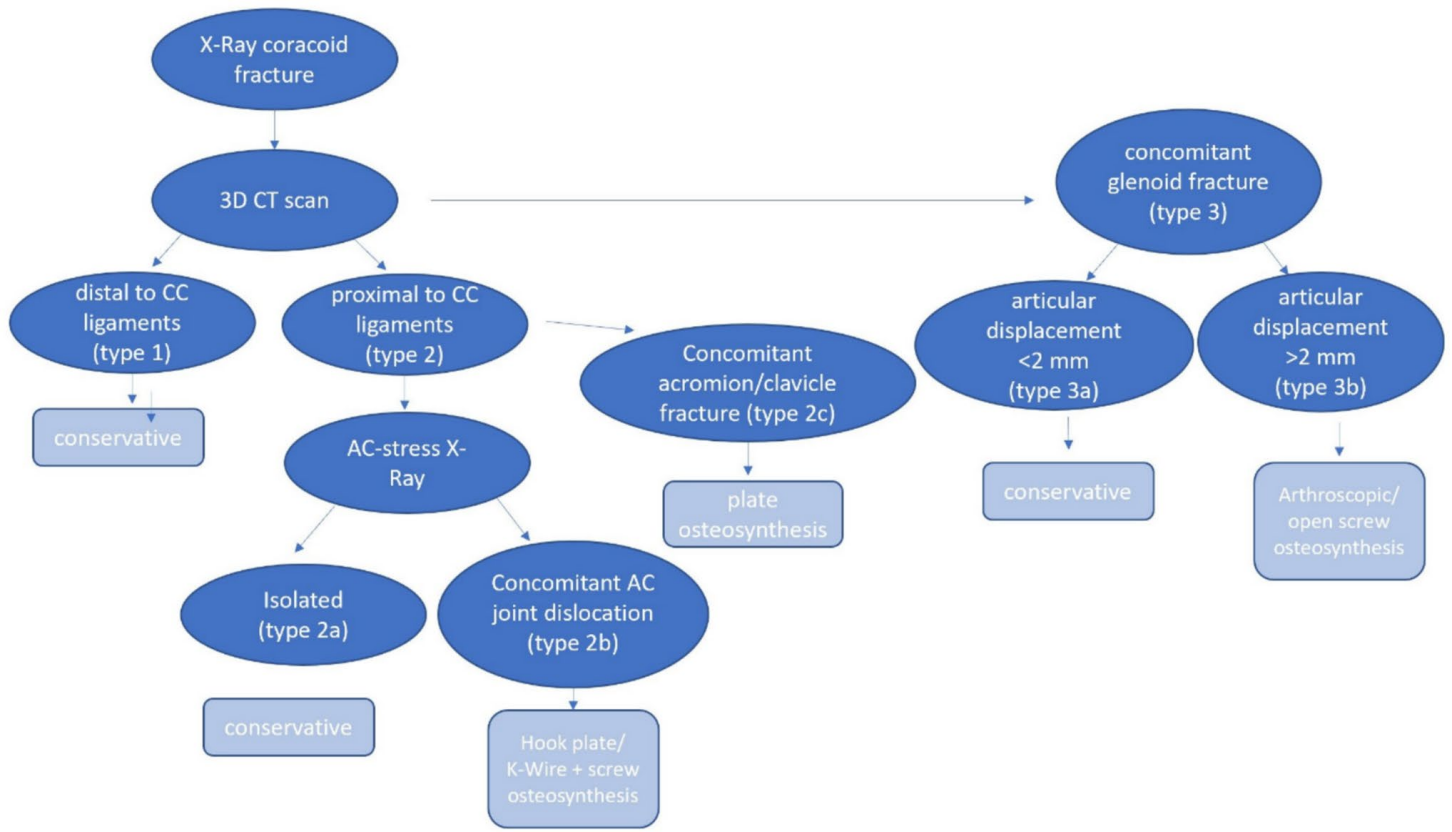
Therapeutic algorithm. CC coracoclavicular; AC acromioclavicular; CT computed tomography;

An overview of our therapeutic algorithm can be seen in Fig. 4.

This study has some major limitations. Firstly, sample is very small. Considering that coracoid fractures are very rare, 15 cases might be a good sample size, but not representative of the general population. Secondly, our groups are inhomogeneous as there were only 4 surgically treated patients but 11 with conservative treatment. Furthermore, all cases had concomitant injuries that might bias outcome results. Additionally, the mean age of the groups differed of 19 years. Therefore, a balanced comparison is not possible. To establish profound knowledge, further studies with larger sample sizes are mandatory. Our presented classification system is a suggestion, but would also need more cases for validation.

## Conclusion

Coracoid fractures are rare lesions which often affect young men with high energy trauma. Concomitant injuries are common and affect most patients. Coracoid fractures may easily be overlooked and if a coracoid fracture is suspected or in case of unclear shoulder pain after trauma, CT imaging is essential to being able to detect such fractures. Depending on the fracture morphology and on concomitant injuries, surgical or conservative treatment can be recommended. This study shows that, if indicated, both surgical and conservative treatment lead to good long-term results. In order to choose the right treatment option, all patients with coracoid base fractures should be assessed with an additional Zanca stress radiograph to detect an associated ACJ dislocation representing a second lesion to the SSSC. We describe 6 different fracture types and recommend paying particular attention to glenoid involvement and multiple lesions of the SSSC. In coracoid fractures with concomitant ACJ dislocation, acromion or clavicle fracture and coracoid fractures affecting the glenoid fossa with a displacement of more than 2 mm, surgical treatment should be considered.

## Data Availability

No datasets were generated or analysed during the current study.
